# Triplicate Dynamic Cell Culture Platform for Enhanced Reproducibility in Anti-Cancer Drug Testing

**DOI:** 10.1021/acsbiomaterials.4c02142

**Published:** 2025-01-14

**Authors:** Yu-Lun Lu, Chiao-Min Lin, Jen-Huang Huang

**Affiliations:** Department of Chemical Engineering, National Tsing Hua University, Hsinchu 30013, Taiwan

**Keywords:** dynamic cell culture, drug cytotoxicity testing, microfluidics, reproducibility, organ-on-a-chip

## Abstract

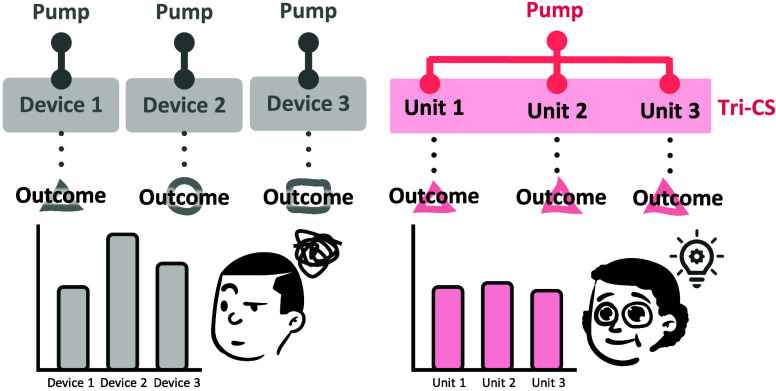

The development of stable and standardized in vitro cytotoxicity testing models is essential for drug discovery and personalized medicine. Microfluidic technologies, recognized for their small size, reduced reagent consumption, and control over experimental variables, have gained considerable attention. However, challenges associated with external pumps, particularly inconsistencies between individual pumping systems, have limited the real-world application of cancer-on-a-chip technology. This study introduces a novel triplicate cell culture system (Tri-CS) that simultaneously supports dynamic cultures in three independent units using a single peristaltic pump, ensuring consistent flow conditions. Our findings demonstrate that the Tri-CS significantly reduces variability compared to individual pump systems, enhancing the reliability of anticancer drug cytotoxicity testing. Furthermore, we evaluated gemcitabine cytotoxicity, which shows enhanced drug efficacy in dynamic conditions. Fluorescein diffusion tests revealed greater diffusion efficiency in dynamic cultures, which contributed to the higher observed drug efficacy. The potential for broader application of the Tri-CS, including its compatibility with commercially available transwells and the opportunity for use in more complex cancer-on-chip models, positions this system as a valuable tool for advancing microphysiological systems in preclinical research.

## 1 Introduction

In preclinical research, *in vitro* models are commonly used to evaluate drug efficacy and safety, especially in large-scale, preliminary screenings, forming an essential part of early drug development processes. The results from these models help identify promising drugs for further investment.^1^ Among these, conventional two-dimensional (2D) cell cultures on standard plates are the most common due to their high throughput, low cost, and fewer variables, which make results more reproducible. However, these models are overly simplistic and fail to replicate complex physiological environments.^2^ To better mimic in vivo conditions, many technologies have advanced toward dynamic culture systems, leading to organ-on-a-chip (OoC) technology, or cancer-on-chip (CoC) in cancer research.^3^ These systems allow for or three-dimensional (3D) cultures with fluid perfusion, simulating blood or tissue fluid dynamics. CoC has gained broad application for modeling disease, aiding in drug development, and personalized medicine by evaluating treatment efficacy. Due to its higher accuracy compared to traditional models, CoC technology enables more precise predictions of drug responses in vivo, making it widely adopted in various cancer studies.^4^

Existing CoC systems often incorporate perfusion mechanisms to sustain cell characteristics and simulate cancer pathologies like circulating tumor cell migration during metastasis.^5^ The circulatory fluid system, in particular, has garnered attention for replicating circulatory, metabolic, and pharmacokinetic processes, while reducing media and drug consumption through fluid recirculation.^6^ Most circulation systems rely on external pumps for fluid control, with peristaltic pumps being a common choice. These pumps connect the media reservoir to the chip, enabling long-term dynamic cultures. However, as with many pumps, peristaltic pumps face low throughput, with most supporting only one or two chips simultaneously.^7,8^ For drug efficacy tests, three or more biological replicates for each experimental condition are typically needed to ensure reliable data.^9^ Moreover, performing these replicates simultaneously helps reduce variability from biological samples and operational conditions.^10^

Multichannel peristaltic pumps have been developed to support multiple units from a single device, theoretically providing a streamlined solution.^11^ However, challenges such as excessive size and overheating make them unsuitable for use in incubators. Alternatively, using multiple pumps to handle different chips allows for simultaneous experiments but often results in inconsistencies. Minor variations between pumps can significantly impact fluid flow, particularly in OoC or CoC systems, where precision is crucial due to the small fluid volumes and slow flow rates.^8^ At the microscale, various physical forces and mass transport processes play a role in delivering biochemical and mechanical signals, such as cytokines and shear stress. These subtle differences can directly affect cellular behaviors, including metabolism, signaling, and differentiation.^12^ Pretesting and calibrating multiple pumps add complexity and time to experiments, further increasing the likelihood of introducing variables in cell or drug handling. Additionally, external pumps require tubing to couple the fluid into the chip, increasing the need for manual handling, the risk of leaks, dead volumes, and nonspecific binding of small molecules to surfaces. While custom high-throughput pumps are expensive and difficult to develop, it remains challenging for emerging OoC and CoC technologies to secure reliable preliminary data.^12,13^

To address the issues posed by external pumps, numerous miniaturized pumps have been developed for integration into biological chips, including impedance-based pumps,^14^ magnetically driven fluid pumps,^15^ and pneumatically controlled pumps.^16^ However, existing technologies each have their drawbacks, such as the inability to drive multiple culture units in parallel under the same conditions, a heavy reliance on customized rather than commercial components, or the necessity for large external equipment like air compressors, gas cylinders, and control systems. Simplifying operations, including the setup of equipment, while minimizing spatial requirements and enabling simultaneous multiple experiments under identical conditions remains a significant obstacle.

We present a triplicate cell culture system (Tri-CS) that uses a single peristaltic pump to maintain three identical dynamic culture conditions. It consists of two circuits: water and medium circulation, separated by a poly(dimethylsiloxane) (PDMS) membrane in the micropump. The peristaltic pump drives water flow through silicone tubing, powering the micropumps for medium circulation, resembling the heart and pulse connected by blood vessels. In previous studies, we demonstrated a dual culture system (Dual-CS) that generated identical fluid conditions for two cell cultures.^17^ Here, Tri-CS represents an enhanced iteration of the Dual-CS, incorporating three key improvements: (i) improved fluid stability by modifying the water flow to a bidirectional circulation, which ensures more consistent medium flow and reduces fluctuations, (ii) a redesigned culture area that accommodates standard transwells, widely used in biological research, thus enhancing flexibility and making the system more versatile for various experimental setups, and (iii) high reproducibility, allowing for consistent and reliable results across multiple experiments, crucial for drug testing and cell culture studies. These advancements make it a valuable tool for dynamic cell culture experiments, particularly in drug testing and biological research.

## 2 Materials and Methods

### 2.1 Device Fabrication

The Tri-CS system was constructed using a layer-by-layer stacking method. Each polymeric sheet layer was meticulously designed using Solid Edge 2D software (ST9, Siemens PLM Software, Germany), and detailed 2D designs of the Tri-CS system are illustrated in Supporting Figure 1. Designs specific to the Microcheck valves can be found in Supporting Figure 2. Before device fabrication, the polymeric sheets were prelaminated with biocompatible adhesive tapes (9122, 3 M Company) on one or both sides. The sheets were then cut using a CO_2_ laser cutting machine (PLS6.75, Universal Laser System), with the laser settings provided in Supporting Table 1. The device was assembled by carefully stacking each layer using an alignment jig, followed by lamination with a seam roller to ensure proper adhesion and to prevent air bubble formation and fluid leakage.

For the fabrication of the PDMS membrane used in both the Microcheck valve and micropump, PDMS membranes were prepared by mixing Sylgard 184 Silicon Elastomer A (prepolymer) (SYGARD-184AB, Goal Bio, Taiwan) and Sylgard 184 Silicon Elastomer B (curing agent) (S184B, Goal Bio, Taiwan) in a 10:1 weight ratio. The mixture was stirred and degassed under vacuum until no visible air bubbles remained. The degassed solution was then spin-coated onto a 0.1 mm PET disk affixed to a 1.5 mm PMMA plate. The spin-coating process was carried out at 1000 rpm (rpm) for 40 s, followed by a 2-s deceleration. After coating, the plates were cured in an oven at 70 °C for 24 h. The resulting PDMS membrane, with a thickness of 85 μm, was used for further experiments. To improve the membrane’s elasticity and functionality, it was laminated between PET and acrylic sheets using the same layer-by-layer stacking method.

**Figure 1:**
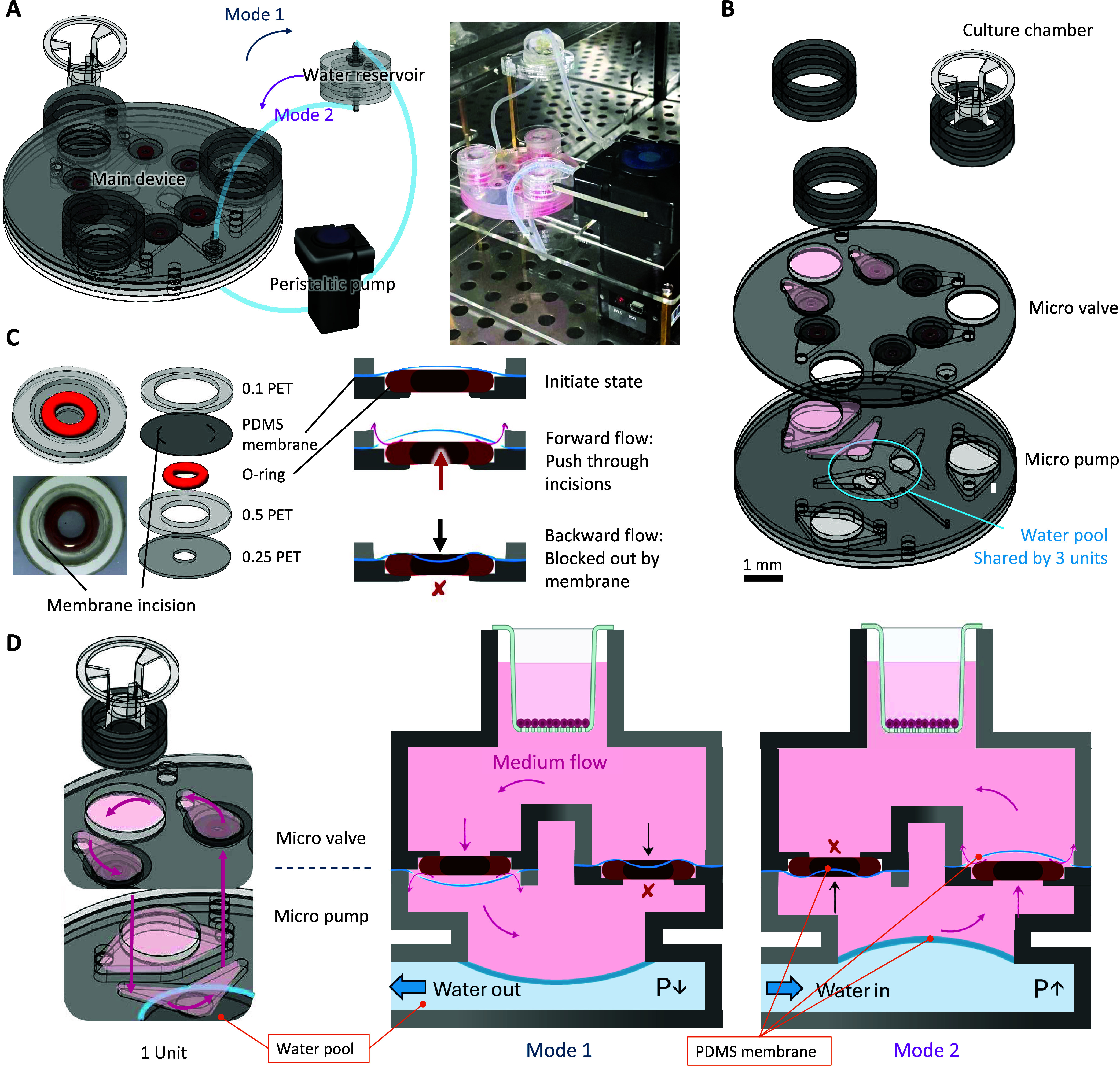
Structural diagram and operating principle of the Tri-CS system. (A) The overall composition and a photo of the Tri-CS system, which consists of a culture chip, a peristaltic pump, and an external water reservoir, all connected to form a closed-loop system. (B) The detailed structure of the culture chip in the Tri-CS system, vertically divided into three layers: the micro pump, the micro valve, and the culture chamber. The water pool in the bottom layer of the micro pump is shared by the three units. The pink-shaded area represents the region where the medium is present. Scale bar = 1 mm (C) The micro valve consists of an O-ring and a PDMS membrane with openings. The design ensures unidirectional fluid flow. (D) Schematic of a single unit and operating modes of the culture chip. A unit is composed of a micro pump connected to the shared water pool, two micro valves, and a culture chamber. The pink arrows indicate the direction of medium flow. In Mode 1, the PDMS membrane in the micro pump moves downward due to lower pressure in the water layer, driving the medium above to flow in through the left valve. In Mode 2, the PDMS membrane in the micro pump moves upward due to higher pressure in the water layer, pushing the medium out through the right valve. Both micro valves contain PDMS membranes with openings, arranged in conjunction with an O-ring to function as unidirectional valves.

In the design of the Microcheck valve, an O-ring (YANG CHENG PACKING Corporation, Taiwan) was incorporated to enhance the valve mechanism. Two materials, nitrile butadiene rubber (NBR) and silicone, were chosen for the O-ring. The microvalve itself has a diameter of 8 mm, with the O-ring measuring 1 mm for the inner diameter (I.D.) and 2 mm for the outer diameter (O.D.). Additionally, small incisions (2.8 mm in diameter) were made in the PDMS membrane, slightly larger than the O-ring (2 mm O.D.), ensuring the membrane tightly attaches to the O-ring when there is a backward flow. These incisions enable the valve to function as a unidirectional opening, allowing fluid to pass in only one direction. The detailed design and working principle are shown in Figure 1C.

All components within the Tri-CS system are reusable. These components are assembled using biocompatible adhesive tapes and are easy to detach from each other. This design facilitates detailed sterilization and allows for convenient replacement of parts, reducing manufacturing costs. The dimensions and functions of all system components are detailed in Supporting Table 2.

### 2.2 Device Sterilization

Upon completion of the device fabrication, the components were subjected to sterilization. Due to the susceptibility of PMMA to breakage upon exposure to ethanol, alternative sterilization methods were employed. Specifically, the main device, holding shelves, silicone tubings, and microvalves were sterilized separately before being assembled within a biological safety cabinet (BSC). Sterilization was performed using the CoolCLAVE Plus Ozone and UV Sterilizer (E330110, Genlantis), which utilizes ozone, UV light, and heat for effective sterilization. Each sterilization cycle lasted approximately 30 min and was conducted using a mode that combined ozone and UV treatment. To ensure thorough sterilization, each set of devices underwent this process at least twice. Additionally, the outer water reservoir, peristaltic pump, and loading shelf were sterilized in the BSC using UV light for a duration of 5 min.

### 2.3 System Setup

The Tri-CS system consists of a peristaltic pump with 4 rollers (Body, DMX-J-SA-17, ARCUS; head, Renze Fluidic, China), a device, and an outer water reservoir, all interconnected by silicone tubings (ULTRA-C-062–1F, Sani-Tech). Two sections of silicone tubing connected to the peristaltic pump have an I.D. of 1.6 mm, while the tubing linking the outer reservoir to the device has an I.D. of 0.8 mm. This difference in inner diameter results in varying flow velocities within the water cycle, with the smaller-diameter tubing functioning as a regulating mechanism.

The setup procedure for the Tri-CS platform begins with the installation of microvalves into the main device, which are then covered to create a closed micropump system that is subsequently sterilized. Following this, the water cycle is established by connecting the peristaltic pump, outer reservoir, and device with the silicone tubings. The reservoirs are filled with water, and the pump is activated to verify the normal operation of the micropumps, as indicated by consistent membrane movements. After ensuring proper flow, culture medium is added to each unit, and the system is washed twice to ensure cleanliness (Supporting Movie 1). The holding shelves were then installed, followed by the insertion of the transwells (35024, SPL Insert Hanging, Korea). A PDMS lid was placed on top of the holding shelves to cover the culture chamber. Finally, the assembled platform is placed into an incubator to maintain the desired experimental conditions.

### 2.4 Flow Pattern, Flow Rate Detection and Shear Stress Calculations

The programmable peristaltic pump was configured using computer software (Arcus Technology) to execute forward rotation for 2 s followed by backward rotation for 1 s, generating a periodic flow within the Tri-CS system. To monitor the flow pattern in real time, a liquid flow meter (SLI-2000, Sensirion) was connected downstream of the Tri-CS, allowing for measurement and assessment of the flow patterns and stability.

To measure the flow rates of the system, the silicone tubing connected to the device was directed into a collecting reservoir for 3 min, and the collected volume was measured using a pipet. The stability test was conducted by operating the platform at rotation speeds of 20, 30, and 60 rpm over five consecutive days. Flow rates from three units were recorded three times a day. Daily average flow rates were calculated to establish the relationship between unit flow rates and the different rotation speeds.

Additionally, the flow rates of three peristaltic pumps were measured to verify system performance. Factors such as the dimensions and materials of the silicone tubing influenced the flow outcomes. In this study, silicone tubing with an O.D. of 3.2 mm and an I.D. of 1.6 mm was used. The measurement locations of the system for the flow pattern and flow rate are as shown in Supporting Figure 2. Flow rates from the three peristaltic pumps were measured by collecting the flow volume at rotation speeds ranging from 3 to 11 rpm, with measurements taken over a 1 min interval.

In our dynamic culture system, the shear stress exerted on the bottom of the Transwell was calculated to ensure that it remains within a range that would not adversely affect cell viability. Using the formula , where μ is the dynamic viscosity of the medium (approximately 1 cP at 37 °C), *Q* is the volumetric medium flow rate (458 mm^3^/min at a pump rotation rate of 20 rpm), *W* is the width (the diameter of the culture chamber, 13 mm), and *H* is the thickness of the liquid layer (3.25 mm).

### 2.5 Cell Culture

The A549 cell line (60074, Bioresource Collection and Research Centre) was maintained in high-glucose Dulbecco’s Modified Eagle’s Medium (DMEM, SH30022.01, HyClone, GE Healthcare Life Sciences) supplemented with 10% bovine calf serum (SH3007203HI, HyClone, GE Healthcare Life Sciences) and 1% penicillin/streptomycin solution (SV30010, HyClone, GE Healthcare Life Sciences) for 3 days. When the cells reached approximately 80% confluence, they were detached using 0.25% trypsin-EDTA (SH30042.01, HyClone, GE Healthcare Life Sciences) and subsequently seeded onto transwells. The transwells were placed in a 24-well plate and cultured in an incubator to allow uniform attachment of the cells to the membrane before being transferred into the Tri-CS system.

### 2.6 Cell Viability and Drug Cytotoxicity Analysis

Cell viability was assessed using the Live/Dead Viability Assay (04511, Sigma-Aldrich). The staining solution consisted of 1 μL calcein-AM (excitation/emission (Ex/Em) = 495:635 nm, GFP for live cells) and 2 μL ethidium homodimer-1 (Ex/Em = 485:530 nm, RFP for dead cells), both dissolved in 1 mL of phosphate-buffered saline (PBS). After removing the medium from each well, 30 μL of the staining solution was added, and the samples were incubated at 37 °C with 5% CO_2_ for at least 15 min. Cell images were captured using the CELENAS Digital Imaging System (Logos Biosystems, Korea). The obtained images were analyzed using the ImageJ software (version 1.53, NIH). In simple terms, the mean fluorescence intensity of the GFP and RFP channels was measured separately. The GFP signal intensity was then divided by the sum of the GFP and RFP signal intensities to calculate the cell viability of each well.

For drug treatment quantitative analysis, the cytotoxicity was measured using the Cell Counting Kit-8 (CCK-8) (CK04–05, Dojindo). To account for variations in evaporated volume, all medium was removed before adding 100 μL of fresh medium and 10 μL of the CCK-8 reagent, maintaining the recommended 10:1 ratio. The cells were incubated for 1.5 h, after which 100 μL of the solution was transferred to a 96-well plate, and absorbance was measured at 450 nm using a multiplate reader (Multiskan EX, Thermo Fisher Scientific). The resulting absorbance values were normalized by the average absorbance of the control group.

To evaluate in vitro drug cytotoxicity, Gemcitabine (HY-17026, MedChemExpress) was used. The drug was initially dissolved in dimethyl sulfoxide (DMSO) (SI-D2650, Sigma-Aldrich) to a stock concentration of 30 mM. This solution was then diluted in DMEM medium to a final concentration of 30 μM, ensuring the DMSO concentration in the medium remained at 0.1%. Medium containing either the drug or vehicle control was added to the culture chambers or wells after allowing the cells to attach to the bottom during a 24-h preincubation. After a 2-day incubation with or without the drug, cell viability tests were performed to assess the effects of the treatment.

### 2.7 Small Molecules Diffusion Analysis

Fluorescein (46955, Sigma-Aldrich) was introduced into the circulation system for the dynamic groups and into the wells for the static groups. Basal medium was added to the insets, and the entire platform was placed in an incubator. At specific time points (4, 8, 12, and 24 h), 5 μL of medium was collected from the insets using a pipet and diluted to 100 μL in a 96-well plate. Absorbance at 490 nm was then measured using a microplate reader.

The Tri-CS system was operated at 20 rpm, while the Single-CS system was operated at 3.5 rpm. Fluorescein solutions with concentrations ranging from 100 to 3% were prepared to establish a dilution curve. For the experiment, 100% fluorescein solution was added to both the circulation system (dynamic group) and the wells of the static system. The dilution curve, which demonstrated a linear relationship between fluorescein concentration and optical density (OD) values (*R*^2^ > 0.99), was used to calculate the fluorescein concentrations in the experimental groups based on their measured OD values. These calculated concentrations were then compared to assess the diffusion behavior in both systems.

### 2.8 Statistical Analysis

Statistical analyses were conducted using GraphPad Prism 8 (GraphPad Software). Differences between individual groups were evaluated using Student’s *t* tests or one-way ANOVA. Data were normally distributed, and group variances were comparable. Statistical significance was indicated by *p*-values of <0.05, < 0.01, and <0.001, corresponding to one, two, and three asterisks, respectively.

## 3 Results

### 3.1 System Concept Description

The entire Tri-CS system is composed of a culture chip, a peristaltic pump, and an external water reservoir, all connected to form a closed-loop configuration (Figure 1A). The culture chip itself can be subdivided into three layers: the micro pump, the micro valve, and the culture chamber, which houses the transwell. The detailed diagram and exploded views of the layers are illustrated in Supporting Figure 1. Notably, although the system accommodates three independently functioning units, they share the same water pool in the bottom side of micro pump layer (Figure 1B). This water pool is connected to an external peristaltic pump, which creates instantaneous pressure differences by drawing water in or out of the pool. These pressure changes cause the PDMS membrane in the micro pump to oscillate vertically, driving the medium above the membrane in the three units. The medium layer contains two unidirectional micro valves with opposite flow directions, preventing backflow and maintaining a consistent one-way flow of the medium. Micro valve consists of an O-ring and a PDMS membrane with curved openings on two sides. The relative positioning of these two components determines the direction of fluid flow (Figure 1C). Specifically, in Mode 1, water is extracted from the pool, lowering the pressure and causing the membrane to move downward, drawing the medium downward. Since one valve has the O-ring positioned above the PDMS membrane, the medium flows through the O-ring and continues through the openings on the membrane. For the other valve, however, the PDMS membrane is positioned above the O-ring, so when the medium flows downward, it is first blocked by the PDMS membrane, and the openings in the membrane are sealed by the O-ring below, preventing the medium from passing through. Conversely, in Mode 2, water is introduced into the pool, increasing the pressure and pushing the membrane upward, forcing the medium upward. As previously mentioned, due to the structural differences between the two valves, the medium can only flow out through the right valve. By continuously alternating between Mode 1 and Mode 2, a unidirectional circulation of the medium is established in the upper layer (Figure 1D).

### 3.2 Stability of the Tri-CS under Different Speeds and Prolonged Operation

**Figure 2:**
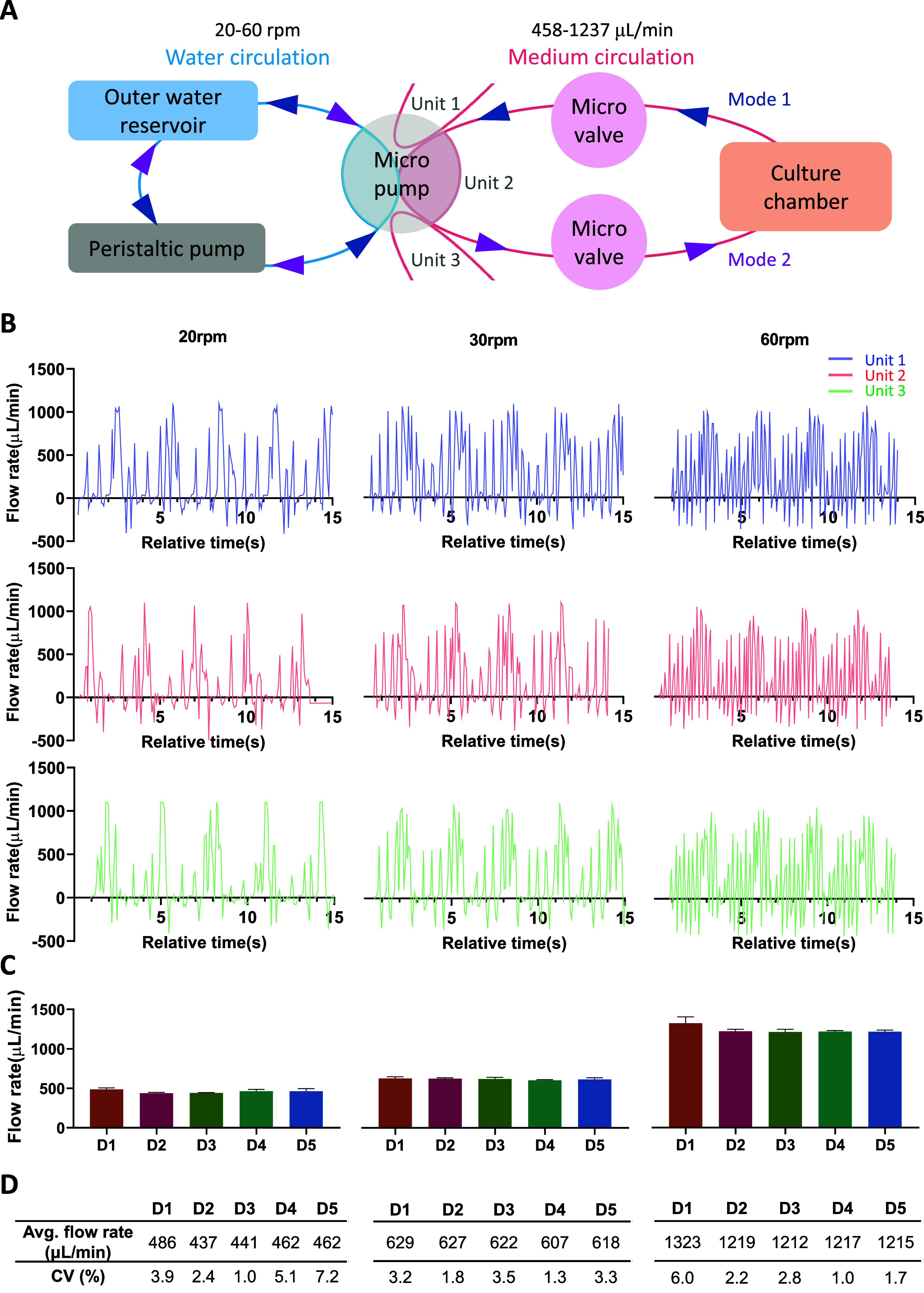
Medium flow stability between units in the Tri-CS system. (A) Working concept of a single unit. The water cycle is maintained via silicone tubing, allowing medium circulation within the device. The two different arrow colors indicate the direction of fluid flow in two distinct modes. External pump speeds ranging from 20 to 60 rpm result in internal medium flow rates between 458 and 1237 μL/min. (B) Flow patterns of the three units at external peristaltic pump speeds of 20, 30, and 60 rpm. Units are represented from top to bottom in blue, red, and green, corresponding to Unit 1, Unit 2, and Unit 3, respectively. (C) Average flow rates of the three units over 5 days at the three different flow speeds. Error bars represent the standard deviation (SD), with *n* = 3. (D) CV calculated for the three units over 5 days at different flow speeds.

The Tri-CS system is engineered to address substantial variability in biological research data by utilizing a single peristaltic pump to maintain consistency across the three units simultaneously. The operational concept is depicted in Figure 2A, which elucidates the relationship between the external pump and the medium flow within the system. First, we examined the medium flow patterns within the system. Figure 2B shows the medium flow patterns of the three units at different external peristaltic pump speeds, revealing consistent and nearly synchronized pulsatile flow across all units. A comparison of medium flow patterns at three different pump speeds revealed that higher speeds resulted in more frequent pulses, indicating a corresponding increase in flow rate within the device. We then recorded the flow rate of the medium within the Tri-CS chip at various external pump speeds. In addition to collecting data from all three units, we also monitored the system over a continuous five-day period (Figure 2C). The results showed that regardless of whether the pump was running at high or low speeds, the flow rate differences between the units remained minimal over 5 days of operation. The average flow rates within the chip were 458, 621, and 1237 μL/min at pump speeds of 20, 30, and 60 rpm, respectively, resulting in shear stresses ranging from 3.13 × 10^–4^ to 9.04 × 10^–4^ Pa. These values are significantly below the thresholds known to influence cellular morphology or function.^18^ Furthermore, the coefficient of variation (CV) was calculated across different pump speeds and time points, with an average variation of 3.1% over the five-day period (Figure 2D). These results indicate that the Tri-CS system maintains stability within an external pump speed range of 20–60 rpm over 5 days, ensuring consistent medium flow patterns across all three units, providing a reliable environment for long-term cell culture experiments.

### 3.3 Comparison of Flow Rate Stability Between Tri-CS and Individual Peristaltic Pumps

**Figure 3:**
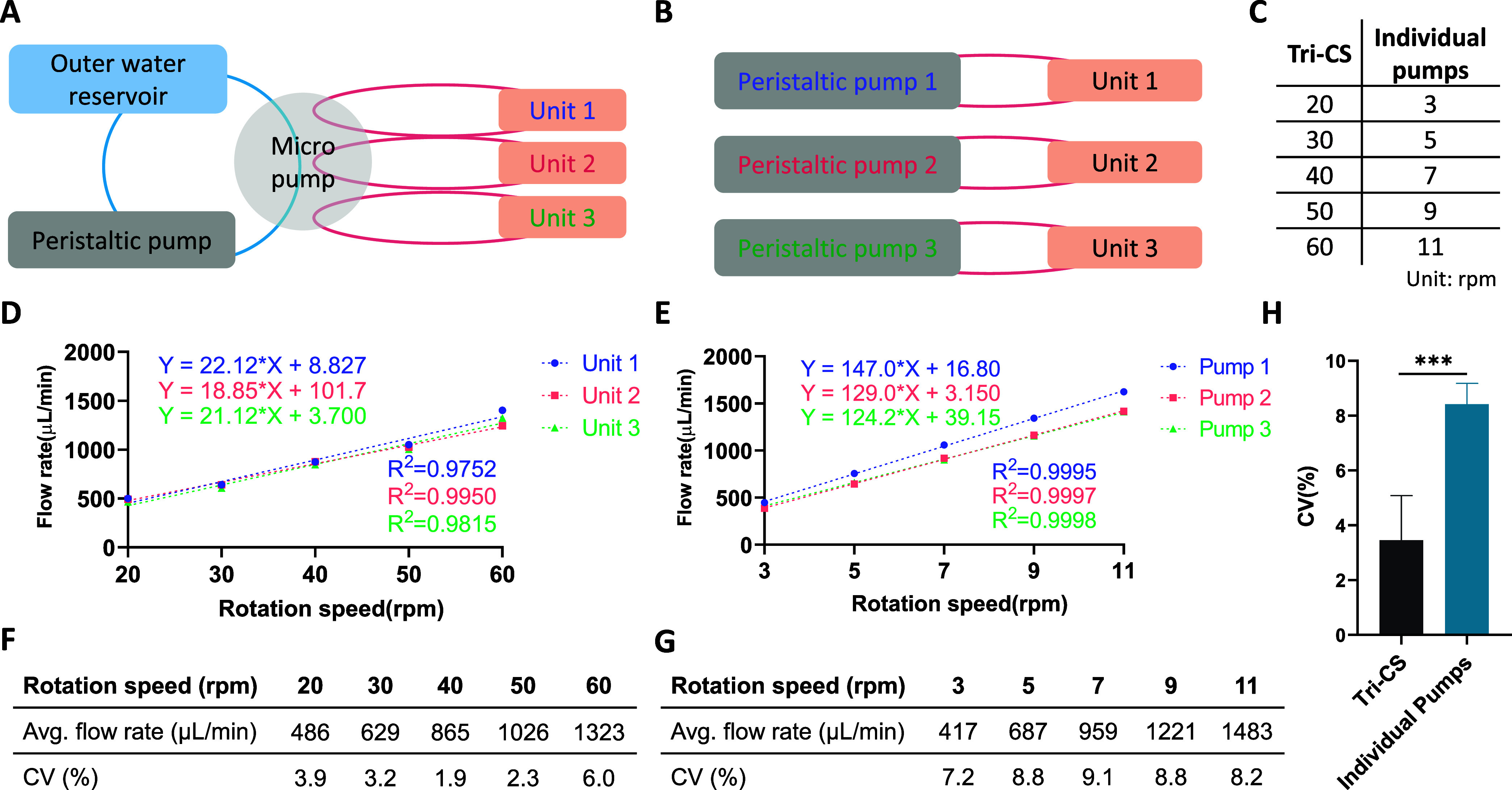
Medium flow rate stability comparison between Tri-CS and individual pump Systems (A) Schematic of three units driven by the Tri-CS system. (B) Schematic of three units driven by individual pumps. (C) Table of adjusted pump speeds for individual pumps to achieve similar medium flow rates as in the Tri-CS system. (D) Comparison of the relationship between pump speed and medium flow rate across different units in the Tri-CS system, with each unit represented by a different color. (E) Comparison of the relationship between pump speed and medium flow rate across different pumps, with each pump represented by a different color. (F) Average flow rate and CV of different units in the Tri-CS system at five pump speeds (20, 30, 40, 50, 60 rpm). (G) Average flow rate and CV of different pumps at five pump speeds (3, 5, 7, 9, 11 rpm). (H) Comparison of the CV between the two methods, showing that the CV for Tri-CS averaged 3.5% across five pump speeds, while the average CV for the individual pump system was 8.4%, which was significantly higher than that of Tri-CS. *P*-values less than 0.001 are indicated by three asterisks (***). Error bars represent the SD, with *n* = 3.

Tri-CS utilizes a single peristaltic pump to drive three units simultaneously, minimizing errors that could arise from using separate mechanical pumps. To highlight this advantage, we compared the flow rate stability between Tri-CS and three individual peristaltic pumps. In this comparison, each pump directly controlled its respective unit, with no shared components (Figure 3A,B). Five different pump speeds were set for both systems. Since Tri-CS uses a micro pump that divides the auxiliary water flow to drive three units simultaneously, the medium flow rate within each unit is slower compared to the direct pump-driven flow from individual peristaltic pumps at the same pump speed. To create comparable flow conditions, the individual peristaltic pump speeds were adjusted to 3, 5, 7, 9, and 11 rpm to match the medium flow rates in Tri-CS, which were set to 20, 30, 40, 50, and 60 rpm, respectively (Figure 3C).

The results demonstrated that in both the Tri-CS and the individual pump system, the medium flow rates in each unit exhibited a strong linear correlation with the pump speeds, as indicated by *R*^2^ values greater than 0.9 (Figure 3D,E). This relationship indicates that both the micro pumps in the Tri-CS system and the individual peristaltic pumps operate steadily across different speeds, allowing for predictable medium flow rates in each unit or pump. Upon examining the linear plots more closely, we observed that the flow rates between the units in the Tri-CS system were remarkably similar, with CV values ranging from 2–6% (Figure 3F). This low variability suggests that the Tri-CS system effectively ensures uniformity in medium flow rates among its units, which is critical for experimental consistency and accuracy.

In contrast, the flow rates among the individual pumps varied significantly at the same speed, with CV values ranging from 7–9% (Figure 3G), resulting in an average CV of 8.4%, which was markedly higher than the 3.5% observed in the Tri-CS system (Figure 3H). This higher variability indicates that the individual pump system is prone to discrepancies between units, which could potentially lead to inconsistencies in experimental results. These findings highlight the advantage of the Tri-CS system in providing more stable and predictable medium flow rates, thereby reducing the risk of errors that can arise from variations in pump performance. Consequently, this enhanced medium flow stability in the Tri-CS system positions it as a more reliable platform for conducting cell experiments that require precise control over medium flow.

### 3.4 Tri-CS Enables Stable Cell Cultivation

**Figure 4:**
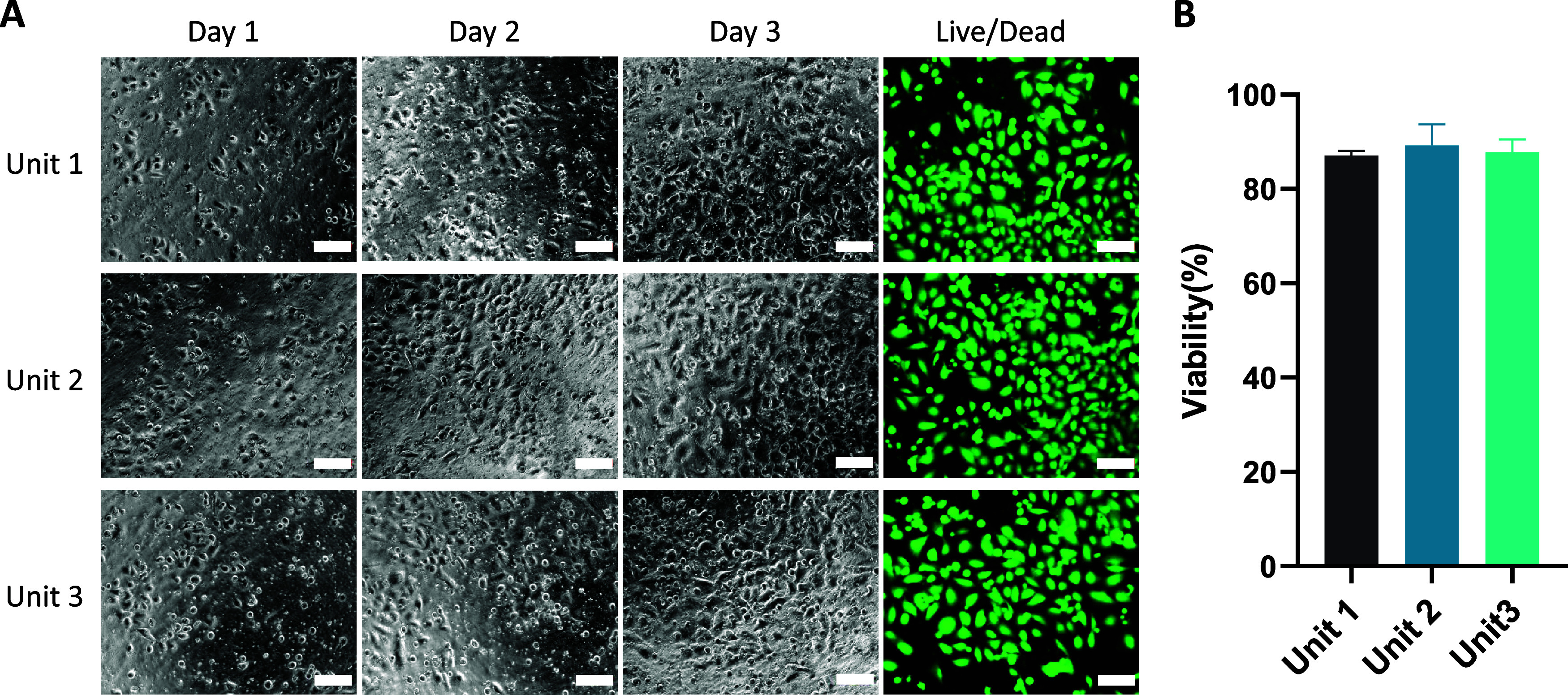
Microscopy images and statistical data of cell viability in the Tri-CS system. (A) Bright-field images and live/dead fluorescent staining on day 3 for A549 cells cultured dynamically in the Tri-CS, with bright-field images taken on days 1, 2, and 3. Scale bar = 100 μm. (B) Statistical analysis of cell viability across three units (*n* = 5). Error bars represent the SD, with *n* = 5.

To assess the feasibility of dynamic cell culture in the Tri-CS system, we evaluated the proliferation and viability of A549 lung cancer cells under a medium flow rate of 496 μL/min, generated by setting the external pump speed to 20 rpm. On day 0, A549 cells were seeded onto transwells and allowed to attach overnight in a 24-well plate under static conditions. The transwells were then transferred into the Tri-CS system for dynamic culture. To facilitate easier observation of cell morphology during the initial 3 days of monitoring, we used a lower seeding density of 8000 cells/well, compared to the higher cell density used in subsequent experiments. The results showed that after 1 day of static culture followed by 2 days of dynamic culture, the cancer cells maintained normal morphology and exhibited steady proliferation throughout the three-day period. There were no significant differences observed among the three units in the microscopy images. (Figure 4A).

To further assess cell viability, we increased the seeding density to 20,000 cells/well, a higher cell number chosen to ensure the reliability of subsequent viability assays by providing a sufficient cell population for accurate analysis of metabolic and proliferative changes under varying conditions. Fluorescence microscopy images on day 3, showing green fluorescence for live cells and red for dead cells, indicated consistent cell proliferation under dynamic culture conditions (Supporting Figure 2). Notably, dead cells were nearly absent, demonstrating high cell survival across all units. The average viability in the dynamic culture was 88%, with a low CV value of only 3.4%, indicating that the performance across the three units was nearly uniform (Figure 4B). These results underscore that the Tri-CS system serves as a reliable platform for cell culture, with its stable fluid flow characteristics providing consistent conditions for cell cultivation across all units.

### 3.5 Impact of Flow Instability and Molecule Diffusion Rate on Drug Efficacy Testing

To highlight the advantages of Tri-CS in enhancing experimental stability, we conducted a comparative study using an alternative system. This alternative, known as the single culture system (Single-CS), operates differently by employing a one-to-one configuration where each unit is driven independently by its own peristaltic pump. Unlike the shared setup of Tri-CS, each pump in Single-CS is solely responsible for a single culture unit composed of a peristaltic pump and a culture chamber containing a transwell (Supporting Figure 3). This section relates to the discussion in Section 3.3, which explained how using individual peristaltic pumps can lead to unstable and unpredictable medium flow rates. Here, we use Single-CS to further investigate the impact of these flow instabilities on drug testing outcomes.

**Figure 5:**
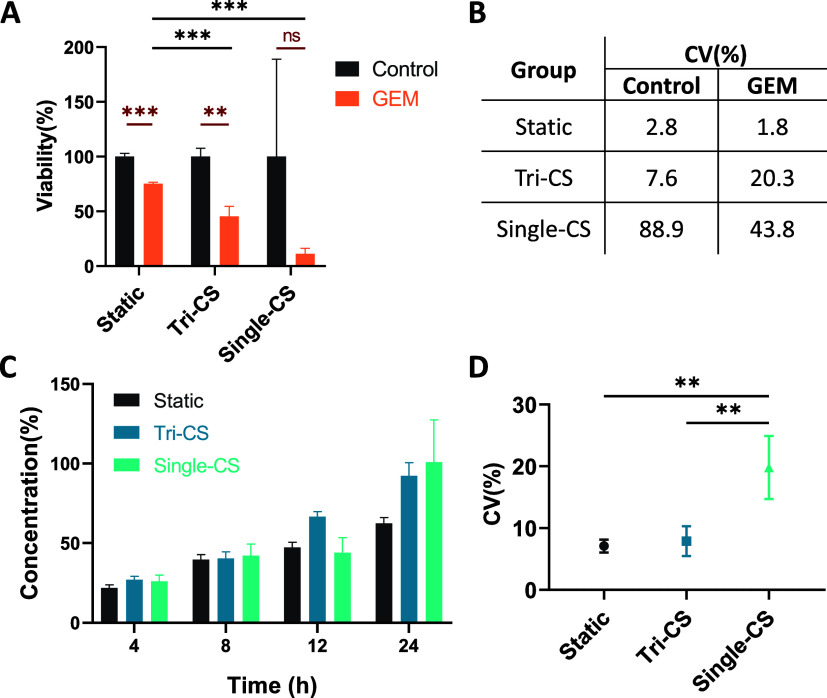
Drug cytotoxicity and diffusion testing in static culture, Tri-CS, and Single-CS systems. (A) Cell viability after treatment with 30 μM gemcitabine for 2 days across all groups. Black asterisks indicate statistically significant differences between groups, while red markers represent independent statistical results within each group. (B) CV values from the drug testing results for each group. (C) The concentration of fluorescence diffusion into the transwell was measured, with data collected at 4, 8, 12, and 24 h. Fluorescent concentration percentages were calculated based on a predetermined standard curve. (D) The CV values from the diffusion testing results showed a significantly higher variation in the Single-CS group compared to the other groups. *P*-values less than 0.001 are indicated by three asterisks (***), and “ns” indicates not significant. Error bars represent the SD, with *n* = 3.

We conducted anticancer drug efficacy tests on both systems to assess their applicability in cytotoxicity testing. The drug used in this study was gemcitabine, a chemotherapeutic agent commonly used in the treatment of nonsmall cell lung cancer. Testing conditions included static culture, where transwells were placed in a well plate, dynamic culture with Tri-CS using a peristaltic pump speed of 20 rpm, and dynamic culture in Single-CS at 3.5 rpm. Both pump speeds were chosen to generate equivalent medium flow rates of approximately 486 μL/min within each system, as described in Section 3.3 (Figure 3A–C). Prior to starting the experiment, cells were cultured overnight in transwells to promote adherence. At the onset of the experiment, the drug was added to the culture chamber or well, rather than directly into the transwell. After 2 days of drug treatment, cell viability decreased across all groups. However, the dynamic culture groups exhibited greater therapeutic efficacy compared to the static group (Figure 5A). Moreover, in the Single-CS system, the CV values were markedly higher in both the control and treatment groups compared to the Tri-CS and static culture groups (Figure 5B). Although the Tri-CS treatment group exhibited a CV of 20%, a statistically significant difference was still detected between the Tri-CS control and treatment groups. Conversely, despite the larger average difference in cell viability between the control and treatment groups in Single-CS, the large variation made it impossible to calculate a significant difference (Figure 5A). These results indicate that the Tri-CS system is highly effective in reducing data variability and improving the likelihood of achieving statistically significant results in experimental treatments.

To further investigate the enhanced therapeutic effect observed in the dynamic culture groups, we analyzed the diffusion dynamics of fluorescein in each experimental group. Since both fluorescein and gemcitabine are small molecules, with molecular weights of 332.31 and 263.198 g/mol, respectively, we hypothesized that the visualization and quantitative analysis of fluorescein could serve as a proxy for the behavior of chemotherapeutic drugs in these groups. Fluorescein was introduced into the culture chambers of each group, and samples were collected from the transwells at various time points (4, 8, 12, and 24 h) for analysis. By measuring fluorescein concentration, the results demonstrated that the diffusion efficiency in the dynamic culture groups was significantly higher than in the static group. This may explain the greater reduction in cell viability in the dynamic groups treated with gemcitabine, as cells in these groups were exposed to higher concentrations of the drug for a longer period (Figure 5C).

Additionally, we calculated the CV values for fluorescein concentrations at each time point across the different groups. The average CV for Single-CS was 19.8%, significantly higher than that of both the Tri-CS (7.9%) and static culture (7.1%). This highlights the instability associated with using individual pumps in Single-CS (Figure 5D). These diffusion test results, when linked to the earlier drug testing outcomes, demonstrate how even minor instabilities in substance distribution, as observed in the fluorescein diffusion (Figure 5C,D), can significantly impact experimental results, such as the larger variability seen in cell viability in the drug treatment experiments (Figure 5A,B). This underscores the importance of maintaining a stable and consistent microfluidic system for reliable biological research outcomes.

## 4 Discussion

A highly stable and standardized in vitro cytotoxicity testing model is crucial for drug development and personalized medicine. Microfluidic technology has garnered significant attention due to its advantages, such as small footprint, minimal reagent consumption, and the ability to control various experimental parameters, making it a promising tool for bridging the gap between traditional cell culture and animal experiments. However, despite the rapid development of biological microfluidic devices in scientific research, these technologies often remain confined to scientific journals, rarely reaching commercialization or widespread application. The journey from promising microfluidic systems to being a practical tool in preclinical research is still ongoing.^13,19^

One of the major challenges in employing microfluidic technology for biological assays is the standardization and long-term stability across multiple units, not just within a single unit.^20^ Additionally, microfluidic systems are often more complex to operate, requiring elaborate setups with multiple tubing connections and devices that increase the demand for technical skill and expertise, which limits the technology’s adaptability across various experimental settings.^13^ Tri-CS provides new insights into fluid-driven microfluidic devices by enhancing throughput without compromising setup time, while ensuring consistency across multiple units. This increases the efficiency of drug screening and reduces interexperimental variability, enabling researchers to obtain comparable results without repeating lengthy and redundant procedures.

Although the current results demonstrate promise in providing stable dynamic culture and drug testing protocols, the long-term stability of the system, particularly beyond 5 days, remains unexplored. Some cancer cell types, including A549 cell line, require extended culture periods to achieve maturity or specific differentiation.^21^ Additionally, the flow rate and flow pattern of the Tri-CS system were measured under cell-free conditions to establish a baseline unaffected by cellular presence. While we acknowledge that the formation of a cell layer can influence flow dynamics, the extent of this impact is highly context-dependent, varying with cell type, density, and experimental conditions. Nevertheless, assessing the impact of cells on flow dynamics is crucial for optimizing platform design and performance. Future studies should incorporate experimental or simulation-based analyses to better understand these interactions and their implications for dynamic cell culture systems. Furthermore, the biological complexity of the present system is still quite low, as it currently focuses solely on 2D cultures. Future research could shift toward utilizing 3D long-term cultured cells to investigate cellular differentiation and functional expression, thereby offering deeper insights into drug testing beyond mere assessments of cell viability.

From an application perspective, Tri-CS is designed to accommodate commercially available transwells, commonly used in scientific research for drug and molecular diffusion assays or for studying cell migration through large-pore membranes. Therefore, beyond serving as a platform for drug testing in fluidic environments, Tri-CS has significant potential as a model for cancer metastasis, such as a metastasis-on-chip,^22^ or for simulating immune cell infiltration into tumor tissues.^23^ Additionally, the water-driven micropump design of Tri-CS provides a new alternative for users who face challenges with throughput when creating recirculation systems using a peristaltic pump. This concept can easily be integrated into various microfluidic devices, potentially advancing the development of microphysiological systems.

## 5 Conclusions

This study developed a novel cell culture system that enables three independent cell culture units to be driven simultaneously by micropumps within a single peristaltic pump-powered microfluidic device. The system is capable of performing drug cytotoxicity testing and, compared to other systems utilizing micropumps, offers easier operation, a smaller footprint, and compatibility with commercially available transwells, enhancing its potential for widespread application. Most importantly, the fluid dynamics between the units are highly consistent, providing greater stability and significantly reducing experimental variation compared to systems using multiple individual pumps. This allows researchers to obtain reliable triplicate data without the need for repeated experiments, saving time and costs. The platform not only accelerates the drug screening process but also has broad application potential, offering new possibilities for the practical use of microphysiological systems in preclinical research.

## Supporting Information Available

The main device design of Tri-CS system, the connection setup for flow measurement, the live/dead images of A549 cells cultured in Tri-CS, the design and operation principle of single-CS, along with tables summarizing the laser cutting conditions, and dimensions and functions of all components in the Tri-CS system (PDF)
Setup and the operation of Tri-CS (MP4)
