# Supplementary material for: Triplicate Dynamic Cell Culture Platform for Enhanced Reproducibility in Anti-Cancer Drug Testing
Source: ACS Biomater Sci Eng. 2025 Jan 14;11(2):1222–31. doi: 10.1021/acsbiomaterials.4c02142 (PMC11815626; doi:10.1021/acsbiomaterials.4c02142)
Supplement: Supplementary file 1 — ab4c02142_si_001.pdf [file ab4c02142_si_001.pdf]

Supporting Information

Triplicate Dynamic Cell Culture Platform for Enhanced Reproducibility in Anti-Cancer Drug Testing

*Yu-Lun Lu[#], Chiao-Min Lin[#], Jen-Huang Huang**

Department of Chemical Engineering, National Tsing Hua University, Hsinchu, 30013 Taiwan

Movie S1. Setup and the operation of Tri-CS.

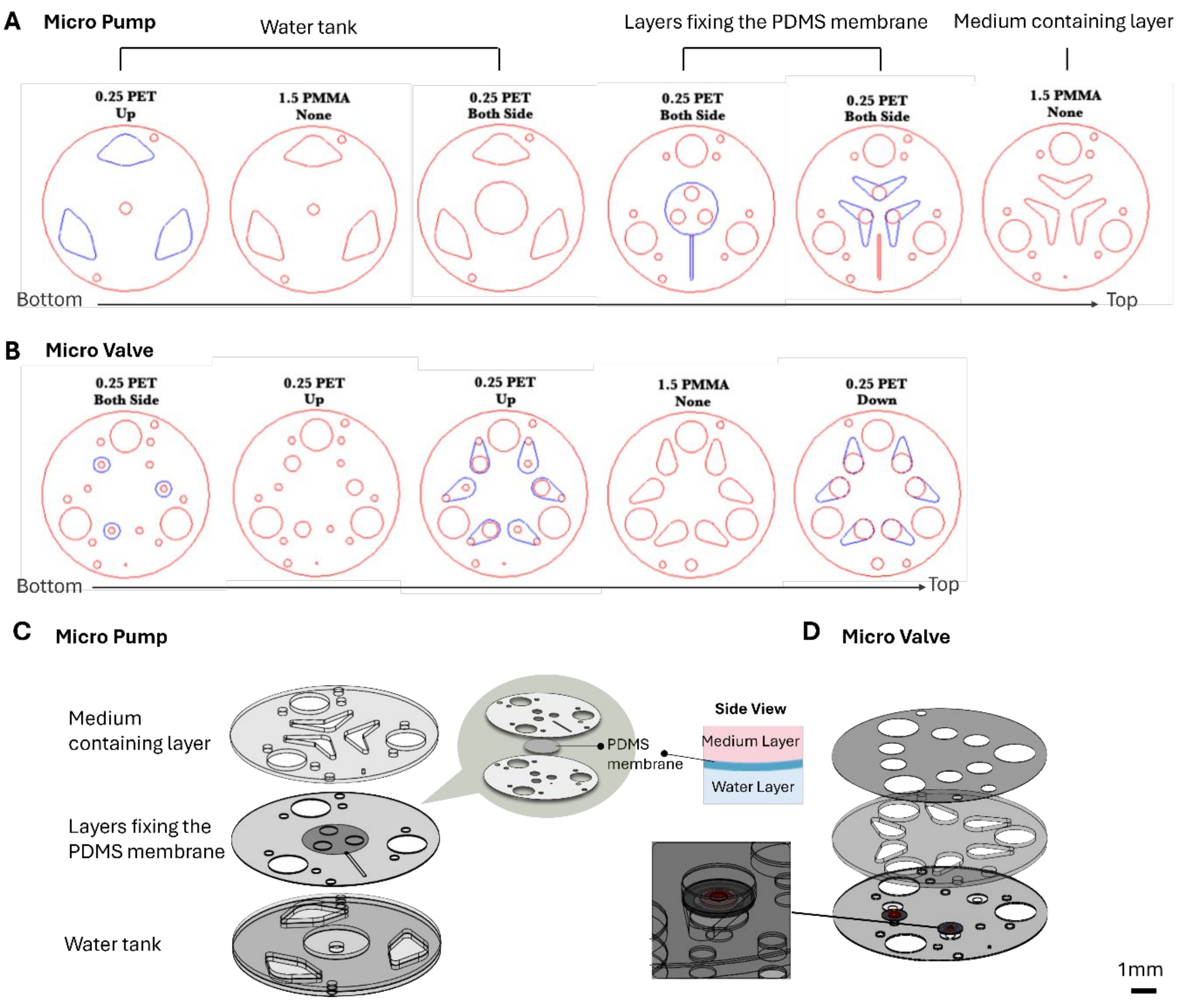

Supplementary Figure 1. Solid Edge diagram of the main device, starting from the bottom layer, showing the materials and the positions of the 3M adhesive tape. Red sections indicate areas where the laser cutter fully cuts through the material, while the blue sections represent areas where only the tape is cut, allowing for easy removal of the adhesive in those regions. **(A)** Diagram of micro pump, including water tank, membrane layer and medium containing layer. **(B)** Diagram of micro valve. "Up," "Down," "None," and "Both side" indicate the position of the 3M adhesive tape on each layer. "Up" refers to the side of the layer facing the upper layer (top), while "Down" refers to the side facing the lower layer (bottom). "None" means that the layer does not have adhesive tape on either side, and "Both side" means that adhesive tape is attached to both sides of the layer. **(C)** Exploded 3D view of micro pump. **(D)** Exploded 3D view of micro valve. Scale bar = 1 mm.

Supplementary Table 1. Optimal parameters for laser cutting under different materials, including cutting powers, speeds, and number of passes.

**Materials**	**Thickness (mm)**	**Tape**	**Power (%)**	**Speed (%)**	**Cutting cycle**
PET	0.1	Both side	65	45	1
	0.25	One side	45	30	1
		Both side	65	25	1
PMMA	1.5	None	75	10	1
	3	None	65	8	3
	4	None	65	10	5
PDMS	0.1	None	50	50	1

Supplementary Table 2. Dimensions and functions of all components in the Tri-CS system

Elements	**Holding shelf**	**Main device**	**Micro valve**	**Culture chamber**
Dimension (diameter × height)	21 mm × 11 mm	68 mm × 9 mm	8 mm × 1 mm	13 mm × 20 mm
Function	To hold the Transwell	Control center where hydraulic force drives medium circulation	To prevent medium flows back	To house the Transwell and medium.

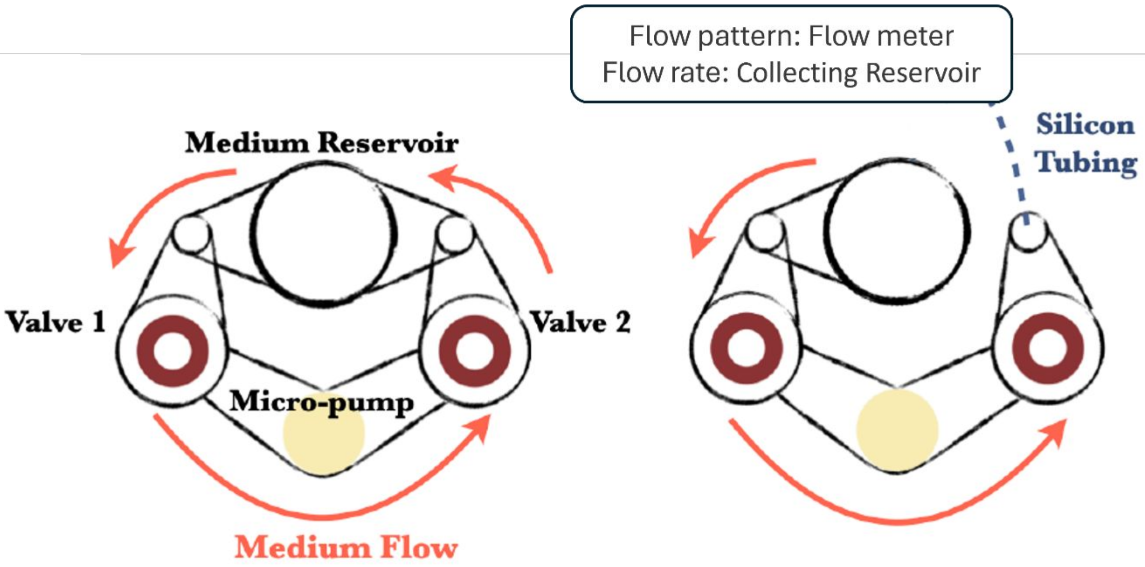

Supplementary Figure 2. Silicone Tube Connection for Flow Measurements. Schematic representation of the original operation diagram (left) and the measurement setup (right), showing the connection to a flow meter for flow pattern measurement and to a collection reservoir for flow rate measurement via external tubing.

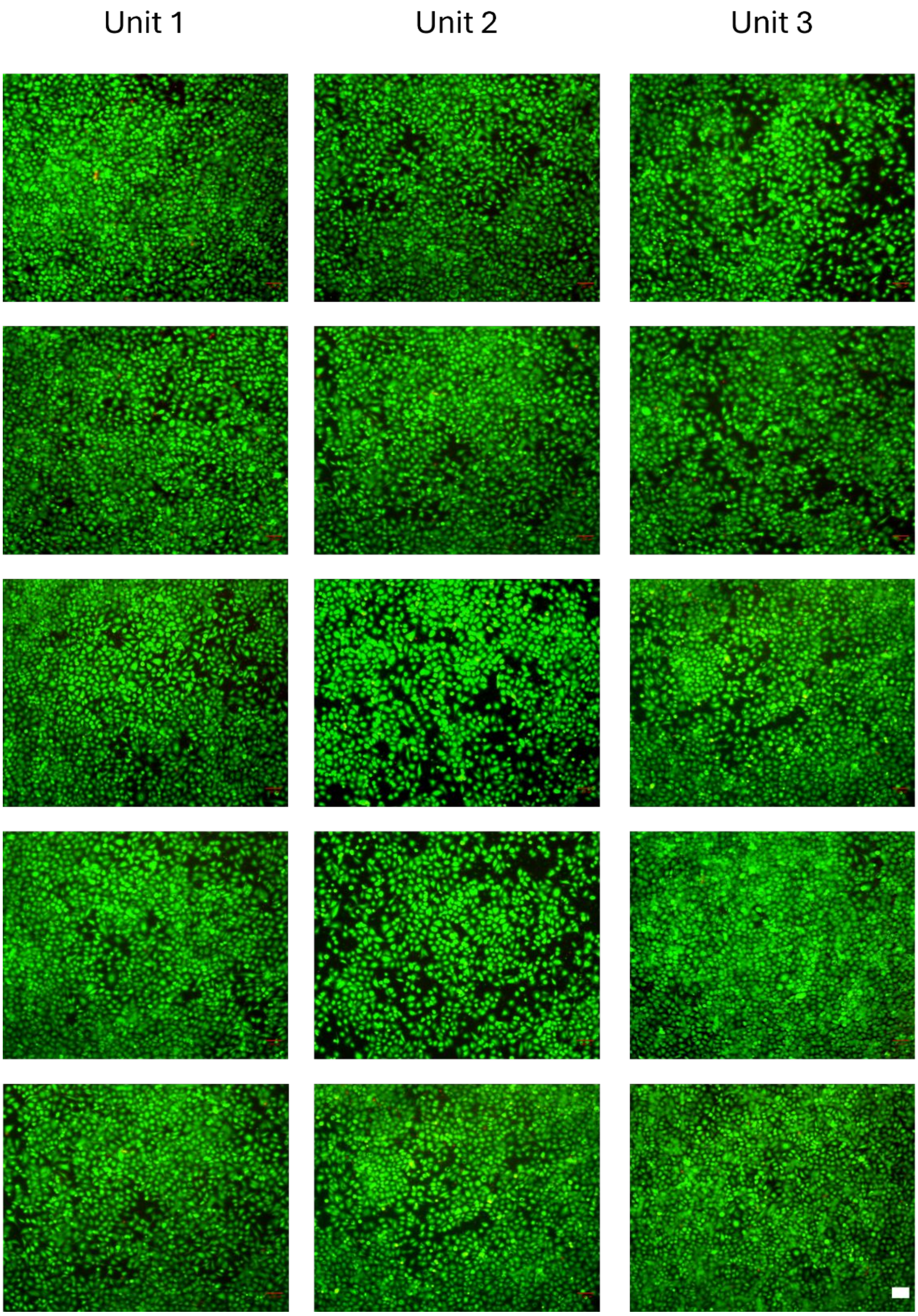

Supplementary Figure 3. Live/dead imaging of A549 cells under dynamic culture in Tri-CS. Fluorescent staining on day 3 shows A549 cells cultured statically for one day to allow attachment, followed by two days of dynamic culture. Green fluorescence marks live cells, while red fluorescence indicates dead cells. Each row represents images taken from different experimental replicates (n = 5), while each column, from left to right, corresponds to units 1, 2, and 3. Scale bar = 100 μm.

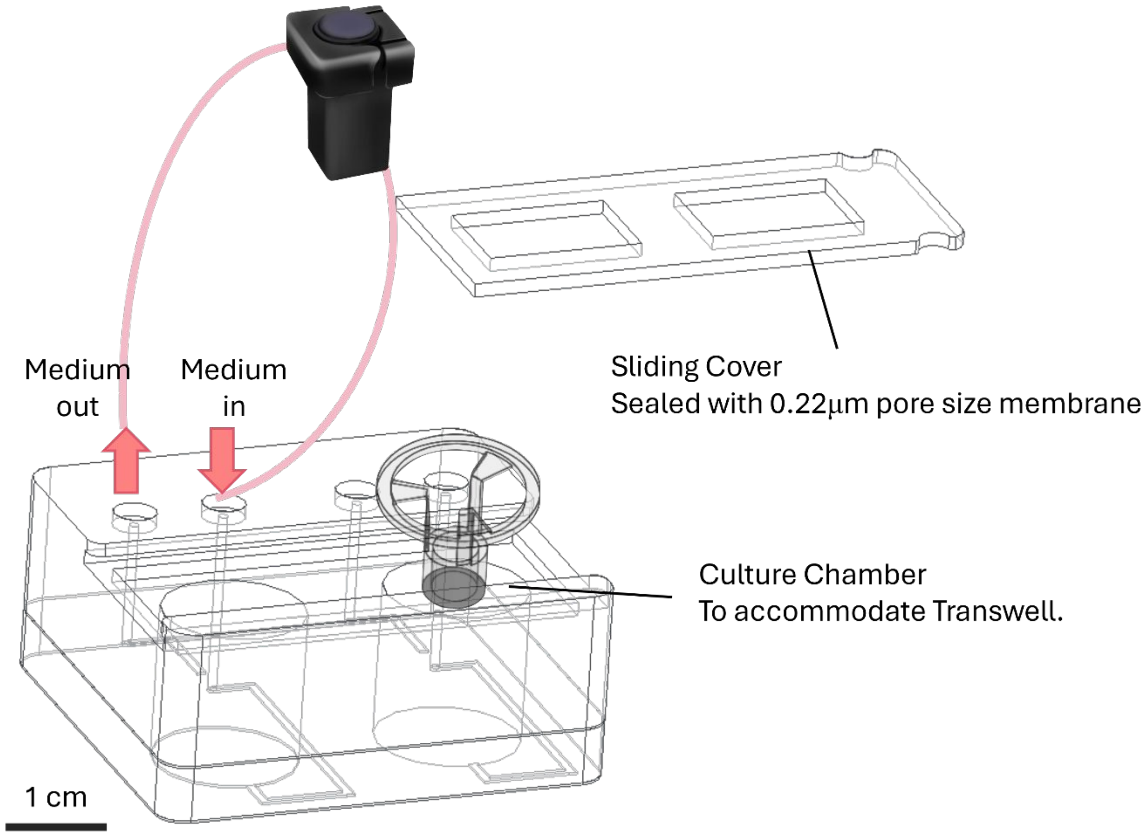

Supplementary Figure 4. Structural diagram and operating principle of single-CS. The system consists of a chip, tubing, and a peristaltic pump. The chip is equipped with a sliding cover, and during operation, a 0.22 µm membrane is affixed to the sliding cover to prevent contamination. Scale bar = 1 cm.
